# Mammalian Eps15 homology domain 1 promotes metastasis in non-small cell lung cancer by inducing epithelial-mesenchymal transition

**DOI:** 10.18632/oncotarget.11220

**Published:** 2016-08-11

**Authors:** Qingwei Meng, Ying Xing, Tingting Ren, Hailing Lu, Yuhui Xi, Zhijun Jiang, Jing Hu, Chunhong Li, Lichun Sun, Dianjun Sun, Li Cai

**Affiliations:** ^1^ The Fourth Department of Medical Oncology, Harbin Medical University Cancer Hospital, Harbin 150040, China; ^2^ The Sixth Department of Medical Oncology, Harbin Medical University Cancer Hospital, Harbin 150040, China; ^3^ Department of Pathophysiology, Harbin Medical University, Harbin 150081, China; ^4^ The Center for Endemic Disease Control, Chinese Center for Disease Control and Prevention, Harbin Medical University, Harbin 150081, China

**Keywords:** EHD1, non-small cell lung cancer, metastasis, epithelial-mesenchymal transition

## Abstract

The identification of the earliest molecular events responsible for the metastatic dissemination of non-small cell lung cancer (NSCLC) remains critical for early detection, prevention, and treatment interventions. In this study, we hypothesized that Mammalian Eps15 homology domain 1 (EHD1) might be responsible for the metastatic behavior of cells in NSCLC. We demonstrated that upregulation of EHD1 is associated with lymph nodes metastasis and unfavorable survival in patients with NSCLC. EHD1 knockdown inhibited the invasion and migration of human NSCLC cells, and overexpression of EHD1 increased the metastatic potential of lung cancer cells. Using the Affymetrix Human Gene 1.0 ST platform, microarray analysis revealed that an association between EHD1 and epithelial-mesenchymal transition (EMT), supported by downregulation of mesenchymal markers and upregulation of epithelial markers following knockdown of EHD1 in cell lines. Moreover, overexpression of EHD1 induced the EMT and increased the metastatic potential of lung cancer cells *in vitro* and *in vivo*. These results provide a model to illustrate the relationship between EHD1 expression and lung cancer metastasis, opening up new avenues for the prognosis and therapy of lung cancer.

## INTRODUCTION

Lung cancer is the leading cause of cancer-related mortality according for about 1.4 million deaths annually [1]. Non-small cell lung cancer (NSCLC) accounts for approximately 80% of all lung cancers, and median survival time of stage I NSCLC is less than 10 years [2]. The main cause of NSCLC related death is the metastasis of cancer cells [3]. Thus, to better understand the mechanisms in metastatic cells is crucial in exploring new anticancer therapy targeting metastasis [4].

The C-terminal Eps15-homology (EH) domain-containing protein 1 (EHD1) is found on the chromosomal band 11q13, and it is an important regulator of many key proteins recycling to the plasma membrane, such as transferrin receptor [5], MHC I [6], MHC II [7], β1 integrin [8] and GLUT4 [9]. For example, one of these important proteins, β1 integrin bind to the extracellular matrix (ECM) and stimulate signaling pathways leading to gene expression, proliferation, cell survival, migration, invasion, metastasis and angiogenesis [10, 11]. Accordingly, we reasoned that EHD1 playing a role in the vesicle trafficking may also be related to cancer invasion and metastasis. The main purpose of this study was to examine a possible function of EHD1 in tumor cell metastasis *in vitro* and *in vivo*.

Metastasis is a complex process, beginning with invasion through endothelial barriers following epithelial-to-mesenchymal transition (EMT), characterized by impaired cell-cell adhesion and increased cell motility [12, 13]. To date, great efforts have been made to understand how EMT is regulated during cancer progression [14–16]. Therefore, identification of biomarkers and investigation of their molecular and biological cellular functions in controlling EMT are important. It is, however, not clear whether EHD1 is involved in promoting EMT in cancer.

The aberrant EHD1 expression has been observed in several malignant tumors including lung, prostate, cervical cancers and cutaneous T-cell lymphoma [17–22]. For example, we have previously identified that EHD1 is a prognostic target related to poor survival in three lung cancer cohorts [17, 18]. Nevertheless, possible function and mechanism of NSCLC metastasis caused by EHD1 overexpression is remain largely unknown.

In the current study, we investigated the expression of EHD1 in 214 NSCLC patients and its relationship with lymph node metastasis and clinical outcome (overall survival [OS], disease-free survival [DFS]). The regulatory mechanism of EHD1 in EMT was investigated in tissues and cell lines by Microarray Processing and Analysis, immunohistochemistry (IHC), Western blot, and cell invasion and migration assay. In addition, we studied the effect of EHD1 protein on metastatic potential in an experimental lung metastasis mouse model.

## RESULTS

### EHD1 expression in NSCLC tissues

**Figure 1:**
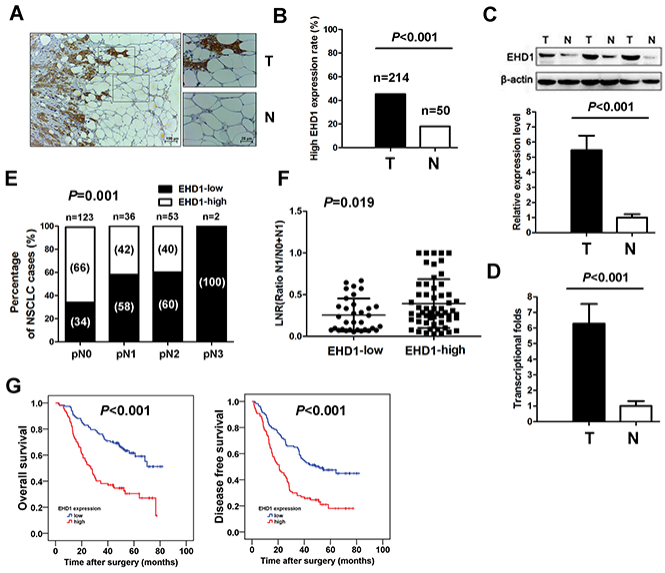
Increased EHD1 expression in NSCLC patients is associated with lymph nodes metastasis and poor survival **A**. Representative immunohistochemistry (IHC) images from a single NSCLC case (T) and matched adjacent normal lung tissue (N). The expression of EHD1 protein in tumor tissues was significantly higher than that in adjacent normal lung tissues. **B**. Histogram showing pooled data derived from NSCLC (T, n=214) and normal lung (N, n=50) tissues. *P* values were calculated using the χ^2^ test. **C**. Representative western blot showing EHD1 expression in lung tissues and a histogram showing pooled data from NSCLC (T, n=20) tissues and adjacent normal lung tissues (N, n=20). **D**. Histogram showing EHD1 mRNA expression in NSCLC (T, n=20) tissues and adjacent normal lung tissue (N, n=20) (right panel). Data are expressed as the mean ± SEM (n = 3). *P* values were calculated using Student's t-test. Normalization: The EHD1/actin ratio was first calculated and normalized to 1.00. **E**. EHD1 overexpression rate in NSCLC with different pN stage. *P* values were calculated using the Fisher exact test. n=number. **F**. Analysis of the lymph node ratio (the ratio of the number of metastatic lymph nodes to the total number of examined lymph nodes) in NSCLC. *P* values were calculated using Student's t-test. **G**. High EHD1 levels are associated with shorter survival in patients with NSCLC. Kaplan–Meier curves showing OS and DFS for patients with high and low EHD1 expression.

IHC analysis revealed that expression of EHD1 protein was significantly higher in tumor tissues than in adjacent normal lung tissues (Figure 1A). In addition, EHD1 expression in NSCLC tissues was significantly higher than that in normal lung tissues (45.3% *vs*. 18.0%, respectively; *P*<0.001; Figure 1B).

We next examined EHD1 protein expression in fresh tumor and normal tissues by western blot analysis. EHD1 was detected as a band of ~61 kDa. The western blotting results showed a higher level of EHD1 protein in NSCLC tissues (n=20) than in normal lung tissues (n=20) (*P*<0.001; Figure 1C).

Expression of EHD1 mRNA was then examined in tumor and normal tissues using real-time quantitative RT-PCR. The results showed that tumor tissues expressed ~6.2-fold more EHD1 mRNA than normal tissues (*P*<0.001; Figure 1D).

### Association of EHD1 expression with patients’ lymph node metastasis and survival

IHC analysis confirmed that EHD1 expression escalated in the pathologic lymph node (pN) classification status from pN0 to pN3 (P=0.001; Figure 1E). We also examined the LNR, which is the ratio of the number of metastatic lymph nodes to the total number of examined lymph nodes [23]. We found that patients with high EHD1 expression had a significantly higher LNR than patients with low EHD1 expression (*P*=0.019; Figure 1F).

Kaplan-Meier analysis demonstrated that high expression of EHD1 predicts a poorer prognosis in terms of both OS (χ^2^= 26.727; *P*<0.001; Figure 1G) and DFS (χ^2^= 21.894; *P*<0.001; Figure 1G). To determine whether EHD1 expression is an independent prognostic factor for overall survival (OS) and/or disease-free survival (DFS) in NSCLC, we performed univariate and multivariate Cox regression analyses (Supplementary Table S1). Advanced pTNM stage and EHD1 overexpression were independent predictors of poor OS and DFS.

### EHD1 overexpression promotes cell motility, migration, and invasion

**Figure 2:**
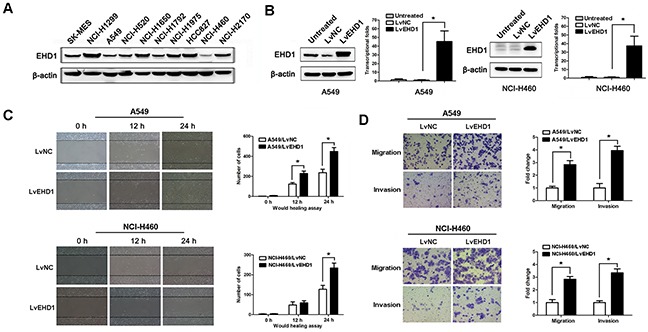
EHD1 increases the motility and invasive properties of non-small cell lung cancer cells **A**. Expression of EHD1 in 10 lung cancer cell lines was examined by western blotting. β-actin was used as a loading control. **B**. Immunoblot and real-time qRT-PCR analysis of EHD1 protein and mRNA expression, respectively, in A549 (left panel) and NCI-H460 (right panel) cells transfected with lentiviruses-EGFP-NC (LvNC) or lentiviruses-EGFP-EHD1 (LvEHD1). *P < 0.05 (Student's t-test). **C**. Wound healing assays were used to examine the migration of A549 (upper panel) and NCI-H460 (lower panel) cells. *P* values were calculated for LvNC versus LvEHD1 using Student's t-test. **D**. The migration and invasion of A549 (upper panel) and NCI-H460 (lower panel) cell lines (and their derivatives) were measured in a Transwell assay. Data are expressed as the mean ± SEM (n = 3). *P < 0.05 for LvNC versus LvEHD1 (Student's t-test).

Western blot analysis detected EHD1 protein in most of the lung cancer cell lines examined (Figure 2A). The human NSCLC cell line A549 and NCI-H460 were chosen as a “gain-of-function” model to further validate the effect of EHD1 on the migratory and invasive behavior of NSCLC.

EHD1 expression was significantly upregulated following transfection of Lv-EGFP-EHD1 (LvEHD1) into A549 or NCI-H460 cells (*P*<0.001; Figure 2B). The wound healing assay results showed that cells transfected with pEHD1 closed scratch wounds more quickly than control cells (*P*=0.029; Figure 2C). Increased EHD1 expression resulted in increased migration and invasion of NSCLC cells when compared with controls (Figure 2D).

### EHD1 knockdown suppresses cell motility, migration, and invasion

**Figure 3:**
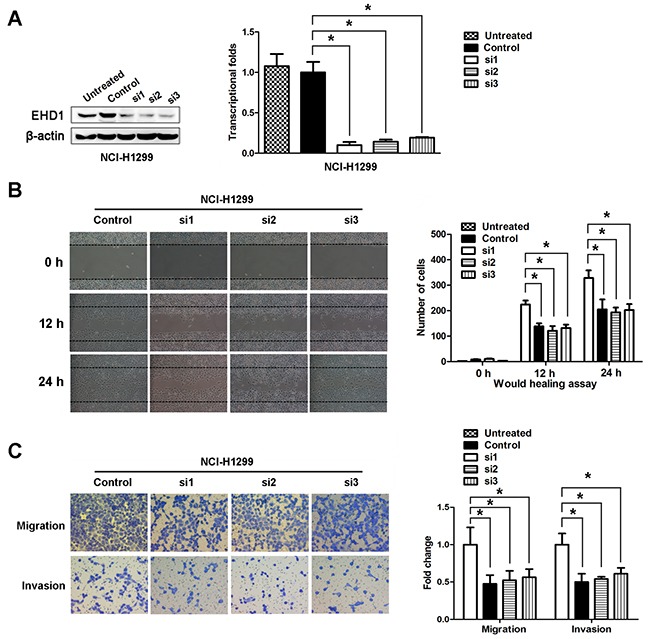
**A**. EHD1 expression was confirmed by immunoblotting and real-time qRT-PCR. EHD1 expression in NCI-H1299 cells was reduced markedly by siRNAs interference. *P < 0.05 (Student's t-test). **B**. Wound healing assays were used to investigate the migration of NCI-H1299 cells. *P* values were calculated using Student's t-test. **C**. Both invasion and migration of NCI-H1299 cell lines (and their derivatives) were measured in a Transwell assay. *P < 0.05 (Student's t-test).

NCI-H1299 cells were selected as a “loss-of-function” model because they expressed high levels of EHD1. As shown in Figure 3, knockdown of EHD1 protein and mRNA at 48 h after transient transfection of a EHD1-specific siRNA was more efficient than after transfection of control siRNA (Figure 3A).

We next used a wound healing assay to test the effects of EHD1 on NSCLC cell motility, migration, and invasion. The results showed that cells transfected with EHD1-specific siRNA were slower to close scratch wounds than control cells (Figure 3B). In addition, a Transwell assay revealed that knocking down EHD1 suppressed NSCLC cell migration and invasion when compared with control cells (Figure 3C).

### Identification of enriched pathways, diseases and functions associated with EHD1 knockdown

**Figure 4:**
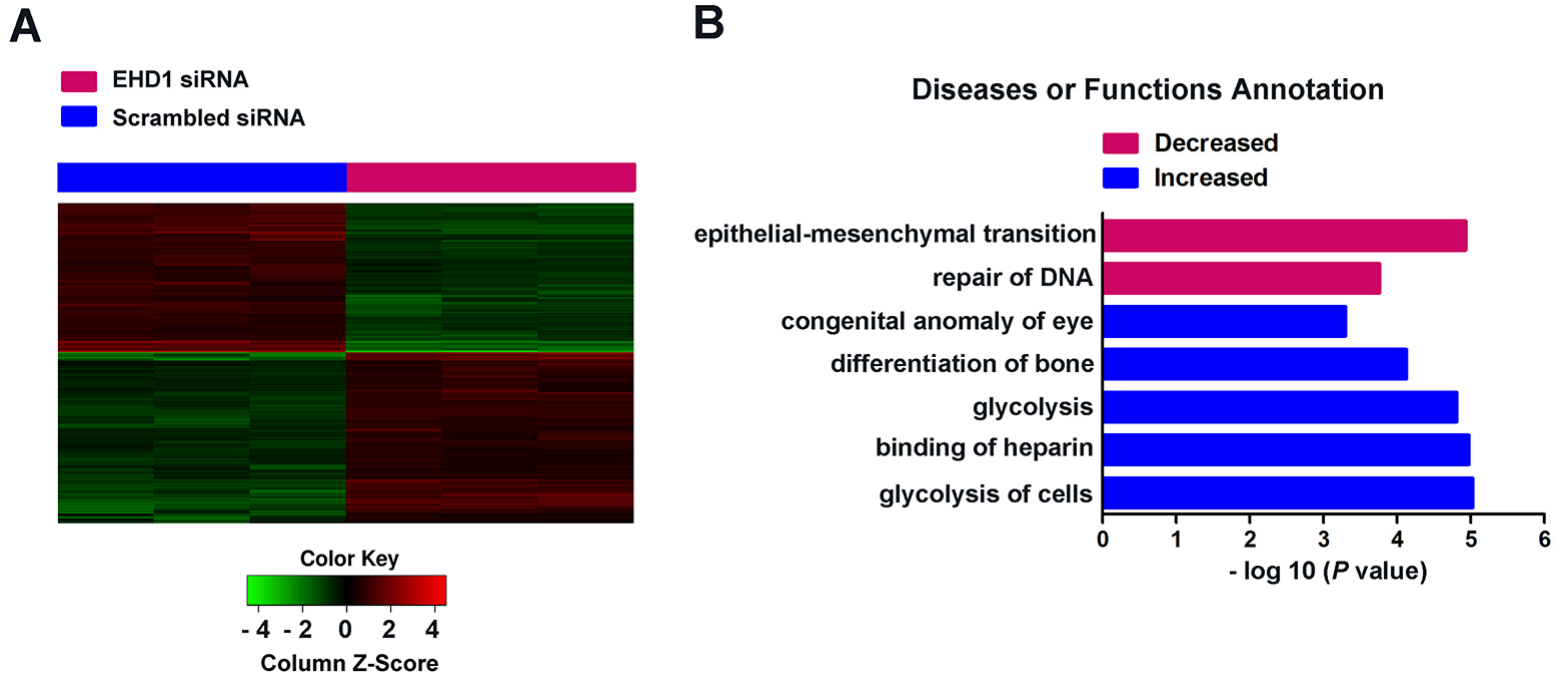
Widespread changes of gene expressions in NCI-H1299 cells with EHD1 knockdown by microarray **A**. Heatmap representation of 582 genes showed significant differential expressions in human malignant lung cancer cell line NCI-H1299 infected with lentivirus expressing either Scr-siRNA (blue) or EHD1-siRNA (red) under the criteria *P*<0.05 and |fold change| >1.3. Genes and samples were listed in rows and columns, respectively. A color scale for the normalized expression data was shown at the bottom of the microarray heatmap (green represents downregulated genes while red represents upregulated genes). **B**. Statistically significant modulation (indicated by the inverse log 10 of *P* values) Diseases or Functions of Annotation following EHD1 knockdown and predicted by the IPA commercially available software is depicted (red represents decreased Diseases or Functions while blue represents increased ones).

Global gene expression profiling of NCI-H1299 cells transfected with either Scr-siRNA or EHD1-siRNA was examined by microarray platform, and significant differential expression was identified in 582 genes (*P*<0.05 and absolute fold change (FC Absolute)>1.3), including 277 upregulated genes and 305 downregulated genes (Figure 4A). Using the IPA commercially available software, we found that EHD1 might affect a wide range of cellular functions by regulating the expression of relevant genes, such as EMT related genes, providing a logical explaination to the higher malignancy in cancer cells with EHD1 overexpression (Figure 4B, Supplementary Table S2).

### EHD1 alters the expression of epithelial and mesenchymal markers

**Figure 5:**
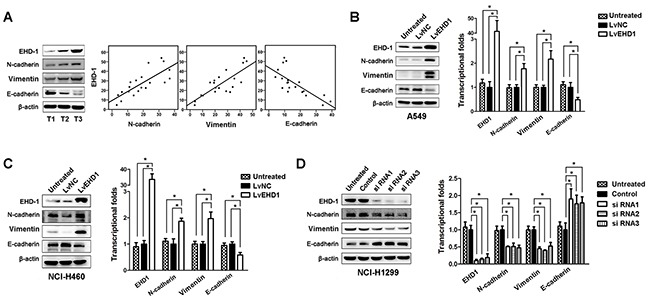
EHD1 promotes NSCLC cell invasion and metastasis by increasing EMT **A**. Representative western blot showing EHD1, E-cadherin, N-cadherin, and Vimentin expression in NSCLC (T, n=20) tissues (left panel). Scatter plot showing the correlation between EHD1 expression and E-cadherin, N-cadherin, and Vimentin expression (right panel). **B, C**. Western blot and real-time qRT-PCR analyses of E-cadherin, N-cadherin, and Vimentin expression in A549 and NCI-H460 cells treated with lentiviruses-EGFP-EHD1 (LvEHD1). *P < 0.05 (Student's t-test). **D**. Western blot and real-time qRT-PCR analyses of E-cadherin, N-cadherin, and Vimentin expression in NCI-1299 cells treated with EHD1-siRNAs. *P < 0.05 (Student's t-test). All experiments were performed in triplicate, with three technical replicates.

Our gene expression profiling analysis showed that EMT was top-decreased following siRNA-mediated EHD1 knockdown in lung cancer cells. To further identify targets regulated by EHD1, we performed western blot analysis of 20 fresh tissue samples to examine the expression of EHD1, N-cadherin, Vimentin, and E-cadherin. EHD1 expression positively correlated withN-cadherin and Vimentin expression, but inversely correlated with E-cadherin expression (Figure 5A). Therefore, we measured the protein levels of E-cadherin, N-cadherin, and Vimentin under conditions of aberrant EHD1 expression. Overexpression of EHD1 inhibited E-cadherin expression and increased Vimentin and N-cadherin expression (Figure 5B–5C). Conversely, knockdown of EHD1 followed a repression of mesenchymal markers, but partially rescued the expression of E-cadherin (Figure 5D). Similar correlations between EHD1 and EMT markers were observed at the transcriptional level (Figure 5B–5D).

### EHD1 overexpression promotes NSCLC cells metastasis and EMT *in vivo*

**Figure 6:**
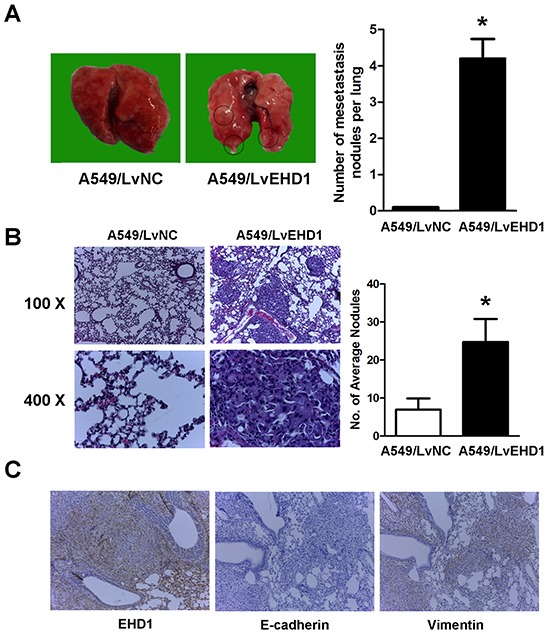
Effect of EHD1 overexpression on metastasis *in vivo* **A**. Representative images of lungs on day 70 after mice were injected with the A549/LvNC and A549/LvEHD1 cells (n = 5 mice per group, left panel). The numbers of metastatic nodules on the surface of the lungs of mice injected with the A549/LvNC and A549/LvEHD1 cells were determined (right panel). Data are presented as the mean and SEM (error bars). *P < 0.05 (Student's t-test). This experiment was repeated three times with similar results. **B**. Representative images of hematoxylin and eosin (H&E) and in lung tissues of mice injected with A549/LvNC and A549/LvEHD1 cells (left panel) are shown. The number of spontaneous lung metastatic lesions in mice was analyzed by counting ten serial sections from each sample (right panel). *P < 0.05 (Student's t-test). This experiment was repeated three times with similar results. **C**. Representative images of immunohistochemical staining of EHD1, E-cadherin and Vimentin in the same tissue of mice injected with A549/LvEHD1 cells.

To further evaluate the effects of EHD1 on the metastasis of lung cancer, the metastatic abilities of A549/LvNC or A549/LvEHD1 were examined in SCID mice (n = 5 per group) via tail vein injection. After 10 weeks, 4 of 5 A549/LvEHD1-injected mice developed metastatic nodules, while none of the mice injected with A549/LvNC cells did. On the surface of the lungs, A549/LvEHD1-injected mice displayed more metastatic nodules than A549/LvNC-injected mice (4.2 per lung *vs*. 0.0 per lung; *P*<0.001), indicative of extravasation to and tumor growth in the lung (Figure 6A). By hematoxylin and eosin staining, lung metastases were observed in all five mice intravenously injected A549/LvEHD1 cells, while much less A549/LvNC-injected mice were found with lung metastases (Figure 6B shows representative hematoxylinand eosin-stained lung sections). The number of spontaneous lung metastatic lesions was counting in ten serial sections from each sample (Figure 6B). To determine *in vivo* the role of EHD1 in EMT induction, IHCconfirmed the relationship between EHD1 and EMT markers expression in the xenograft tissues. From the same xenograft tissue, the tissue section staining strongly for EHD1 also displayed high levels of Vimentin activity (confirmed by staining for Vimentin protein expression), whereas expressed low levels of E-cadherin expression (Figure 6C).

## DISCUSSION

Here, we report that EHD1 expression was significantly increased in NSCLC tissues. Aberrant EHD1 expression has also been reported in other malignancies including prostate cancer, cervical cancer and cutaneous T-cell lymphoma. However, its clinical relevance has not been studied [19–22]. Herein, we demonstrate that increased EHD1 is associated with lymph node metastasis and poor survival. Consistent with this study, we have previously identified that EHD1 is a prognostic indicator related to poor survival in small cell lung cancer or NSCLC [16, 17]. What is the role of EHD1 in cancer progression is intriguing. In the current study, we observed enhanced lung cancer cell motility, metastasis, and invasion induced by EHD1 *in vitro*, and more feasibility for EHD1 overexpressed-cell to forming lung metastasis *in vivo*. Although the results are intriguing, this investigation has limitations. The *in vivo* model we used here to simulate metastasis process is not perfect for lacking of the steps of invasion and intravasation. Thus, a spontaneous lung metastatic model in nude is necessary to design.

In order to uncover the mechanisms underlying EHD1-mediated lung cancer migration and invasion, microarray analysis was performed and 582 genes showed significant differential expression. Furthermore, the gene signatures were examined with functional pathway analysis and multiple pathways involved in cancer progression were unraveled. EMT is the top-decreased function following EHD1 knockdown. EMT is a reversible process during which cells switch from a polarized, epithelial phenotype into a highly motile, mesenchymal phenotype [24]. Playing a key role during both embryonic morphogenesis and wound repair in adult tissue, EMT is now being deemed a critical step during the onset of metastases [25–28]. Based on our microarray analysis result, we speculated EHD1 could act as a regulator of the EMT process in tumor cells. The finding in our study that EHD1 positively correlated with mesenchymal markers, but inversely correlated with epithelial markers *in vitro*, as well as *in vivo*, provides a basis for the study of novel mechanisms underlying EHD1-mediated NSCLC metastasis. However, there might be other mechanisms by which EHD1 induces EMT. Previous functional studies showed that EHD1 modulates β1 integrin function by facilitating recycling of these receptors, and promotes cancer cell migration [8]. It seems that EHD1 plays complex roles during EMT, and more intensive observation is indispensable.

Moreover, analysis of our gene expression data after siRNA knockdown of EHD1 demonstrated it involved various pathways (Supplementary Table S3). For instance, HIPPO signaling is critical for tumor proliferation, migration, and invasion [29, 30]. Other pathways, such as PI3K/AKT Signaling [31], inhibition of matrix metalloproteases [32], and colorectal cancer metastasis signaling, are also closely related to cancer metastasis. These data provided complementary evidence about the causal role of EHD1 in lung cancer cell metastasis. The exploration of possible mechanism of NSCLC metastasis caused by EHD1 overexpression is ongoing in our laboratory.

In light of the above findings, EHD1 can be considered as a novel marker of lymph node metastasis in NSCLC patients. From microarray data, multiple oncogenes and cancer associated pathways were observed to be regulated by EHD1 knockdown, which may be a molecular mechanism underlying lung cancer development and progression. This study not only reveals the pathological role of EHD1 in NSCLC, but also suggests that it could be used as a prognostic factor and therapeutic target in NSCLC and, possibly, other cancers as well.

## MATERIALS AND METHODS

### Clinical specimen and cell lines

Clinical samples were obtained from 214 patients with NSCLC who were surgically treated at Harbin Medical University Cancer Hospital from January 2007 to December 2010. Thirty matched metastatic lymph nodes from 30 patients whose primary tumor expressed high levels of EHD1 were selected for immunohistochemical (IHC) examination. Fresh tissues (paired NSCLC tumor samples and matched adjacent normal tissue samples) were resected from 20 NSCLC patients between June 2013 and June 2014. This study was approved by the Institute Research Medical Ethics Committee of Harbin Medical University. All of the patients gave their informed consent.

The human lung adenocarcinoma cell lines A549, NCI-H1299, NCI-H1975, NCI-H1792, NCI-H1650 and HCC827, the lung squamous cell carcinoma cell lines NCI-H520, NCI-H2170 and SK-MES, and the large cell carcinoma cell line NCI-H460 were purchased from American Type Culture Collection (ATCC, Manassas, VA), which employed short tandem repeat (STR) profiling to ensure cell line authenticity 3 months before the initiation of this study. No other forms of authentication were implemented by the author during the course of the study.

### Immunohistochemistry

Immunohistochemical analysis of EHD1 was performed using the Two-Step IHC Detection Reagent (PV-6001) kit (Zhong Shan Golden Bridge Biological Technology Inc., Beijing, China), according to the manufacturer's instructions. Paraffin-embedded tissue blocks containing lung specimens were cut in a microtome (~4 μm thick) and stained with hematoxylin and eosin (H&E). In brief, tissue sections were deparaffinized in xylene and rehydrated in a series of graded alcohol solutions according to standard procedures. The sections were then immersed in 3% hydrogen peroxide for 10 min to remove endogenous peroxidase. Antigen retrieval was performed for 3 min in a pressure cooker containing 10 mM citrate buffer (pH 6.0) to enhance immunoreactivity.

The slides were then incubated with anti-EHD1 (1:200; Abcam, Cambridge, UK, ab109311), E-cadherin (1:200; ProteinTech, Manchester, UK, 20874-1-AP), Vimentin (1:200; Abcam, Cambridge, UK, ab8978) at 4°C overnight. After washing with phosphate-buffered saline, a rabbit secondary antibody (Zhong Shan Golden Bridge Biological Technology Inc., Beijing, China) was applied and incubated for 20 min at room temperature. Color was developed using 3,3′-diaminobenzidine tetrahydrochloride (Dako, Hamburg, Germany). The slides were then counterstained with Meyer's hematoxylin and dehydrated in ethanol. Finally, the slides were mounted and cover-slipped with Resina. The negative control slides were stained with rabbit serum instead of primary antibodies. All the tissue sections were analyzed by two independent pathologists experienced in evaluating IHC, both of whom were blinded to the clinicopathological data. The staining results were scored according to the following criteria: (a) percentage of immunoreactive cells: 0 (0%), 1 (1–10%), 2 (11–50%), 3 (51–70%), or 4 (≥71%); and (b) staining intensity: 0 (negative staining), 1 (weak staining), 2 (moderate staining), or 3 (intense staining). The final score for EHD1 expression was the sum of both scores; thus, the final score ranged from 0 to 7. For the purposes of statistical analysis, a final staining score of < 4 was defined as low expression, and a score ≥ 4 was defined as high expression. Any discrepancies between scores were reviewed by the two pathologists plus a senior pathologist until a consensus was reached.

### Cell invasion and migration assay

The invasion and migration assays were performed in 24-well FluoroBlok cell culture inserts (BD Biosciences) fitted with a PET membrane (8 μm pore size). The inserts were coated with 100 μL of Matrigel matrix (1 μg/μL; BD Biosciences) at 4°C overnight. Following starvation for 6 h in serum-free RPMI 1640 or DMEM, cells were harvested from a single sub-confluent 10 cm dish using cell dissociation buffer (Life Technologies), spun at 500 × g for 3 min, and resuspended in RPMI 1640. Cells (4 × 10^4^ in 300 μL of RPMI 1640) were then seeded onto the insert and 700 μL of RPMI 1640 supplemented with 10% FBS was added to the lower chamber of each Transwell. After incubating for 18 h at 37°C, the medium inside the insert was removed and the insert placed in a new 24-well plate. The cells present on the reverse side of the insert were then labeled with a fluorescent dye (Calcein AM; 4 μM in Dulbecco's PBS; BD Biosciences) for 1 h at 37°C.

### Microarray processing and analysis

Total RNA from NCI-H1299 cells infected with lentivirus expressing either Scr-siRNA (n=3) or EHD1-siRNA (n=3) was extracted using Trizol reagents. Then RNA quantity and quality were assessed with NanoDrop 2000 and Agilent Bioanalyzer 2100. And Affymetrix human GeneChip primeview was used for microarray processing to determine gene expression profiling depending on the manufacturer's instructions. In brief, reverse transcription, double-stranded DNA template conversion, in vitro transcription for mRNA synthesis and labelling were all

performed using GeneChip 3′ IVT Expression Kit. Microarray hybridization, washing, and staining were then performed using GeneChip Hybridization Wash and Stain Kit. Arrays were then scanned using GeneChip Scanner 3000 to produce raw data. Significant differentially expressed genes between NCI-H1299 cells treated with EHD1-siRNAs and NCI-H1299 cells treated with Scr-siRNAs were selected based on the following criteria: *P* value <0.05 and absolute fold change >1.3. Pathway enrichment analysis was performed for all significant differential genes based on the IPA commercially available software.

### Xenograft models

Healthy purebred BALB/C nude mice were maintained according to the guidelines for the administration of laboratory animal research as outlined by the Institutional Animal Care and Use Committee of Harbin Medical University in China and the Care and Use of Laboratory Animals (National Institutes of Health, revised 1985).

The metastatic ability of A549/LvNC and A549/LvEHD1 was determined following cell injection intravenously into the tail vein. Cells at 2 × 10^6^ cells resuspended in 100 μL phosphate-buffered saline (PBS) were injected into the lateral tail veins of SCID mice (n = 5 mice per group). Metastatic lungs were fixed with paraformaldehyde (4%) before dehydration and paraffin embedding. Paraffin sections were stained with hematoxylin and eosin according to standard protocols or were subjected to IHC staining using a horseradish peroxidase–labeled streptavidin–biotin ABC kit (ZSGBBIO, Beijing, China) with hematoxylin as the counterstain. The research protocol was approved by the institutional ethics committee for the administration of laboratory animals of Harbin Medical University, China.

### Statistical analysis

All analyses were performed using SPSS 19.0 for Windows (SPSS, Chicago, IL, USA). Student's t-test was performed for continuous variables and the χ2 test was used to analyze differences between categorical variables. Survival curves were plotted using the Kaplan–Meier method and compared using the log-rank test. Covariates that remained significant through univariate analysis were selected for multivariate analysis. A two-sided *P* value <0.05 was considered statistically significant.

## SUPPLEMENTARY TABLES






