# Supplementary material for: Mammalian Eps15 homology domain 1 promotes metastasis in non-small cell lung cancer by inducing epithelial-mesenchymal transition
Source: Oncotarget. 2016 Aug 11;8(14):22433–42. doi: 10.18632/oncotarget.11220 (PMC5410234; doi:10.18632/oncotarget.11220)
Supplement: Supplementary file 1 [file oncotarget-08-22433-s001.pdf]

Mammalian Eps15 homology domain 1 promotes metastasis in non-small cell lung cancer by inducing epithelial-mesenchymal transition

SUPPORTING INFORMATION

MATERIALS AND METHODS

Patients and cell cultures

The histological type of lung cancer was defined according to the World Health Organization classifications, and primary cancers were evaluated in accordance with the American Joint Committee on Cancer (7th Ed.) staging system [1]. The study was approved by the Institute Research Medical Ethics Committee of Harbin Medical University. All patients provided informed consent. None of the patients received chemotherapy, radiotherapy, or immunotherapy prior to surgery.

Overall survival (OS) was defined as the time from surgery to the date of death. Disease-free survival (DFS) was defined as the time from surgery to the time of recurrence or death resulting from any cause. All NSCLC patients were followed up with until death or until the cut-off date (December 30, 2014). The mean and median follow-up time after discharge from hospital were 40.1 months and 44.2 months respectively, ranging within 2.2-81.6 months.

Cells were cultured according to the suppliers' instructions. Briefly, NCI-H1650 cells were grown in Dulbecco's modified Eagle's medium (DMEM; Invitrogen, Grand Island, NY) supplemented with 10% fetal bovine serum (FBS; Invitrogen). A549, NCI-H460, and NCI-H1299 cells were cultured in RPMI-1640 medium (Invitrogen) supplemented with 10% FBS (Hangzhou Sijiqing Biological Engineering Materials Co, Ltd, Hangzhou, China). NCI-H1975, NCI-H1792, HCC827, NCI-H520, NCI-H2170 and SK-MES cells were grown in RPMI-1640 medium (Invitrogen) supplemented with 10% FBS (Invitrogen).

RNA interference transfection

Short interfering RNA (siRNAs) sequences specifically targeting human EHD1 were synthesized by Invitrogen (Shanghai, China). The sequences were as follows: siRNA1, CCAAGGUUCACGCCUACAUTTA UGUAGGCGUGAACCUUGGTT; siRNA2, GAAGUG AUCAAGGCUCUGATTUCAGAGCCUUGAUCACU UCTT; siRNA3, GGGAGAGAUCUACCAGAAGTTC UUCUGGUAGAUCUCUCCCTT; non-targeting control siRNA, UUCUCCGAACGUGUCACGUTT.

For siRNA delivery, double-stranded RNA oligonucleotides were transfected using Lipo2000 (Invitrogen). NCI-H1299 (grown to 30–50% confluence in six-well plates) were transfected with siRNAs (75 pmol per well) using 5 μL of Lipo2000 according to the manufacturer's instructions and then harvested 72 hours later for analysis.

Establishment of stable cell lines

For stable transfections, the lentiviruses-EGFP-NC (LvNC) and lentiviruses -EGFP-EHD1 (LvEHD1) vector system were constructed, packed, and purified by GeneChem (Shanghai, China) and manipulated according to the manufacturer's protocol. Briefly, LvNC and LvEHD1 were used to infect A549 and NCI-H460 cells for 3 days. Stable clones were then selected with puromycin (1 μg/mL).

Wound healing assay

Cells were seeded in six-well plates and cultured in RPMI 1640 or DMEM containing 10% FBS until confluent. The cells were then scratched with a sterile 10 μL pipette tip to create artificial wounds. Phase-contrast images of the wound healing process were obtained digitally at 0 and 24 h after wounding using an inverted Olympus IX50 microscope fitted with a 10× objective lens. Eight images per treatment were analyzed to determine average parameters with respect to the position of the migrating cells at the wound edges. Lines were digitally drawn using Image-Pro Plus software (Media Cybernetics, Rockville, USA).

Western blot analysis

Frozen tissue samples or lung cancer cells were homogenized in RIPA buffer containing a 1% protease inhibitor mixture. The mixture was centrifuged at 12 000 g for 15 min at 4°C and the supernatant obtained. Total protein was quantified using the Bradford method (Thermo Scientific, Waltham, MA, USA). Briefly, 30 μg of protein extract was separated by 12% SDS

polyacrylamide gel electrophoresis (SDS-PAGE) and transferred onto PVDF membranes (Millipore Company, Billerica, MA, USA). The membranes were blocked with 2% bovine serum albumin (BSA) at 37°C for 1 h and incubated with the primary antibody overnight at 4°C. After washing, the membrane was incubated with a horseradish peroxidase-labeled secondary antibody for 1 h at room temperature and then washed again. The blots were stained using Super ECL Reagent kit (HaiGene, Harbin, China; M2301) and imaged using the FluorChem™ HD2 System (Proteinsimple, CA, USA). The antibodies used for western blotting are listed in Supplementary Table S4. The experiment was repeated three times.

RNA preparation and reverse transcription

Immediately following resection, fresh tissues were stored at –80°C until RNA extraction. Total RNA was extracted from fresh frozen samples or lung cells using Trizol reagent (Invitrogen, Carlsbad, CA), following confirmation by pathologists that the tumor samples contained at least 75% tumor cells. RNA quality and concentration were measured using a NanoDrop 2000 spectrophotometer (Thermo, Wilmington, DE, USA). Reverse transcription was performed using 2.0 µg total RNA in a 14.5-µL reaction mixture containing 2×Power Taq PCR Master Mix kit (PR1702, BioTeke, Beijing, China), according to the manufacturer's instructions.

Real-time quantitative reverse transcriptase-PCR (qRT-PCR)

Real-time qRT-PCR was performed using the Exicycler96 Real-time RT-PCR system (A-2060, BIONEER, Daejeon, Korea) and SYBR Green Mastermix (SY1020, Solarbio, Beijing, China). The following EHD1 primers were used: forward, 5'-CACTACCGCTTCCACGAGTT-3'; reverse, 5'-GTGTCGGATGAAGGTGGTCT-3'. The following β-actin primers were used as the internal reference: forward, 5'-CTTAGTTGCGTTACACCCTTTCTTG-3'; reverse, 5'-CTGTCACCTTCACCGTTCCAGTTT-3'. Amplification was performed under the following conditions: 95°C for 10 min, followed by 40 cycles of 95°C for 10 s, 60°C for 1 min, and 95°C for 15 s. Experiments were performed in triplicate. The results of the real-time qRT-PCR experiments were calculated using the $2^{-\Delta\Delta C(t)}$ method.

REFERENCES

1. Tsim S, O'Dowd CA, Milroy R, Davidson S. Staging of non-small cell lung cancer (NSCLC): a review. Respiratory Medicine. 2010; 104:1767-1774.

Supplementary Table S1: Univariate and multivariate analyses of overall survival and disease-free survival

Variable	OS			DFS		
	Univariate analysis	Multivariate analysis		Univariate analysis	Multivariate analysis	
	P	HR (95% CI)	*P*	*P*	HR (95% CI)	*P*
Age						
<60						
≥60	0.580	–	–	0.286	–	–
Gender						
Female						
Male	0.683	–	–	0.762	–	–
Differentiation						
Good		Ref				
Moderate		1.245 (0.626 to 1.887)	0.233			
Poor	0.021*	1.667 (0.950 to 1.927)	0.192	0.189	–	–
Histological cell type						
Squamous cell carcinoma						
Adenocarcinoma	0.074	–	–	0.062	–	–
pTNM stage						
I		Ref			Ref	
II		1.601 (1.095 to 2.562)	0.018*		1.366 (1.006 to 1.968)	0.043*
III/IV	<0.001*	2.724 (1.460 to 3.423)	0.004*	<0.001*	2.422 (1.260 to 2.977)	0.005*
Lymph node metastasis						
Present		Ref			Ref	
Absent	<0.001*	1.288 (0.946 to 1.626)	0.166	<0.001*	1.385(0.858 to 1.737)	0.172
EHD1 expression						
Low		Ref			Ref	
High	<0.001*	2.755 (1.382 to 3.461)	0.008*	<0.001*	1.737 (1.240 to 2.433)	0.001*

Abbreviations: NSCLC = non-small cell lung cancer; pTNM stage = Tumor, node, metastasis (pathological stage); p T = pathological T stage; n = number of patients; OS = overall survival; DFS = disease-free survival; HR = hazard ratio; CI = confidence interval. **P*< 0.05 was considered statistically significant.

Supplementary Table S2: Diseases or Functions Annotation.

See Supplementary File 1

Supplementary Table S3: Ingenuity Canonical Pathways.

See Supplementary File 2

Supplementary Table S4: Antibodies used for western blotting

Target	Dilution	Manufacturer
E-cadherin	1:2000	ProteinTech
N-cadherin	1:5000	Abcam
Vimentin	1:5000	Abcam
EHD1	1:1000	Abcam
β-actin	1:1000	Cell Signaling Technology
Mouse secondary antibody	1:10000	Zhong Shan Golden Bridge
Rabbit secondary antibody	1:10000	Zhong Shan Golden Bridge
